# Renal Tissue Oxygenation Monitoring—An Opportunity to Improve Kidney Outcomes in the Vulnerable Neonatal Population

**DOI:** 10.3389/fped.2020.00241

**Published:** 2020-05-14

**Authors:** Matthew W. Harer, Valerie Y. Chock

**Affiliations:** ^1^Division of Neonatology, Department of Pediatrics, University of Wisconsin School of Medicine and Public Health, Madison, WI, United States; ^2^Division of Neonatal and Developmental Medicine, Department of Pediatrics, Stanford University School of Medicine, Palo Alto, CA, United States

**Keywords:** NIRS, tissue oxygenation, kidney, renal, neonatal, preterm, HIE, cardiac

## Abstract

Adequate oxygenation of the kidney is of critical importance in the neonate. Non-invasive monitoring of renal tissue oxygenation using near-infrared spectroscopy (NIRS) is a promising bedside strategy for early detection of circulatory impairment as well as recognition of specific renal injury. As a diagnostic tool, renal NIRS monitoring may allow for earlier interventions to prevent or reduce injury in various clinical scenarios in the neonatal intensive care unit. Multiple studies utilizing NIRS monitoring in preterm and term infants have provided renal tissue oxygenation values at different time points during neonatal hospitalization, and have correlated measures with ultrasound and Doppler flow data. With the establishment of normal values, studies have utilized renal tissue oxygenation monitoring in preterm neonates to predict a hemodynamically significant patent ductus arteriosus, to assess response to potentially nephrotoxic medications, to identify infants with sepsis, and to describe changes after red blood cell transfusions. Other neonatal populations being investigated with renal NIRS monitoring include growth restricted infants, those requiring delivery room resuscitation, infants with congenital heart disease, and neonates undergoing extracorporeal membrane oxygenation. Furthermore, as the recognition of acute kidney injury (AKI) and its associated morbidity and mortality in neonates has increased over the last decade, alternative methods are being investigated to diagnose AKI before changes in serum creatinine or urine output occur. Studies have utilized renal NIRS monitoring to diagnose AKI in specific populations, including neonates with hypoxic ischemic encephalopathy after birth asphyxia and in infants after cardiac bypass surgery. The use of renal tissue oxygenation monitoring to improve renal outcomes has yet to be established, but results of studies published to date suggest that it holds significant promise to function as a real time, early indicator of poor renal perfusion that may help with development of specific treatment protocols to prevent or decrease the severity of AKI.

## Introduction

The use of near infrared spectroscopy (NIRS) to monitor regional tissue oxygenation in neonates was first introduced clinically in the 1980's (1). The mechanism behind the technology has been well-described in previous reviews (1–3). NIRS provides clinicians an estimate of local tissue oxygen utilization by assessing post-capillary oxygenation. Multiple factors may affect NIRS values, but the two main determinants are tissue perfusion and tissue oxygen utilization. In the neonatal intensive care unit (NICU), the principal end-organ clinically monitored with NIRS has been the brain, however, multiple other tissues have been evaluated in neonatal research studies including the kidney, splanchnic circulation and peripheral muscles. In a recent survey of neonatal providers, 61% of respondents used NIRS for monitoring the kidney (4). This survey did not ask what type of patients received renal NIRS monitoring or how this information was used. These survey results highlight the need for specific renal tissue oxygenation monitoring guidelines and protocols to improve neonatal critical care (2).

It has been proposed that specifically in neonates, peripheral NIRS monitoring may be more sensitive to acute changes in oxygenation homeostasis than cerebral monitoring due to the protective physiologic mechanisms that maintain cerebral perfusion (2). This ability to detect sensitive changes in renal tissue oxygenation coupled with a notable increase in neonatal acute kidney injury (AKI) research over the past 20 years has resulted in multiple studies evaluating renal NIRS monitoring in groups of neonates at high risk for kidney injury. With the development of specific neonatal AKI definitions, the epidemiology of kidney injury in neonates cared for in critical care environments has become clear (5, 6). The most accepted current AKI definition is the neonatal modified KDIGO definition (Table 1) (7). Since adopting this definition AKI has been independently associated with increased mortality and length of hospital stay, highlighting the importance of this often overlooked neonatal comorbidity (8, 9). How renal tissue oxygenation monitoring will be used clinically in critically ill neonates to reduce the rates of AKI and improve neonatal renal outcomes is just starting to be understood.

**Table 1:** Neonatal AKI KDIGO definition.

**Stage**	**Serum creatinine**	**UOP over 24 h**
0	No change in sCr or rise <0·3 mg/dL	> 1 mL/kg/hour
1	sCr rise ≥ 0·3 mg/dL within 48 h or sCr rise ≥1·5–1·9 X reference sCr^a^ within 7 days	>0·5 and ≤ 1 mL/kg/hour
2	sCr rise ≥ 2–2·9 X reference sCr^a^	>0·3 and ≤ 0·5 mL/kg/hour
3	sCr rise ≥3 X reference sCr^a^ or sCr ≥ 2·5 mg/dL^b^ *or* Receipt of dialysis	≤ 0·3 mL/kg/hour

a Reference sCr is the lowest prior sCr measurement.

b this is lower than the original KDIGO definition as a sCr of 2.5 mg/dl in neonates suggests a GFR <10 ml/min/1.73m^2^.

*sCr, serum creatinine; UOP, urine output*.

In this review, we attempt to summarize and highlight how clinicians and researchers are using renal NIRS monitoring in neonates in the critical care setting. We include multiple specific groups of neonates that are at high risk for renal abnormalities as well as attempt to define normal values in otherwise healthy neonatal populations. Finally, we propose future directions for research, clinical care and commercial development as it pertains specifically to neonatal renal NIRS monitoring.

## Renal Tissue Oxygenation: How to Monitor and What is Normal?

### Logistics of Monitoring

The first step to monitoring renal tissue oxygenation is selection of a NIRS device and sensor. Although differences in cerebral tissue oxygenation in neonates taken with different devices and probes have been analyzed in previous studies, there have been no published studies that have directly compared renal tissue oxygenation (10). These previous studies show that cerebral tissue oxygenation measures overall correlate well-between devices, but there are differences in the absolute values. A simultaneous comparison of neonatal renal tissue oxygenation levels from multiple NIRS devices would be challenging given limited surface area and underlying differential tissue oxygenation between the left and right kidneys. Evaluation at different time epochs would also be challenging given the inherent variability in oxygenation of the kidneys over short time intervals. Future comparative device studies may benefit from development of an *in vitro* model of the kidneys.

**Figure 1:**
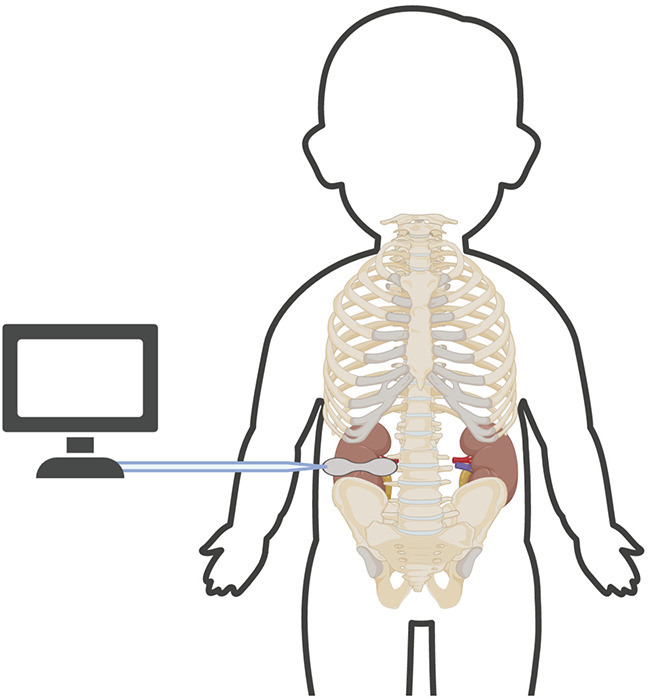
Proper location of neonatal renal NIRS sensor. This image shows the proper placement of neonatal renal NIRS sensor below the costal margin and above the iliac crest with the tip of the sensor lateral to the spine and reading end of the sensor wrapping around the side.

The next step is the appropriate placement of the NIRS sensor. Either kidney may be monitored, and no current studies have evaluated if the right and left kidney have similar tissue oxygenation. The appropriate placement of a renal NIRS sensor is below the costal margin and above the iliac crest with the emitting tip of the sensor lateral to the spine and the reading side of the sensor wrapping around the neonate's flank (Figure 1). Although this is the typical location of the kidney, it is important to note that the NIRS reading in this region is not as precise as cerebral tissue oxygenation readings of the brain. “Renal tissue oxygenation” in this paravertebral region is likely a combination of multiple tissues in the area, including but not limited to fat, muscle and potentially even intestine. With a fixed depth of penetration of near-infrared light dependent on the type of NIRS monitor and size of the sensor, in some very small neonates, readings may not truly be reflective of renal tissue oxygenation. Limited neonatal studies have correlated saturations from renal NIRS monitoring to renal blood flow measures (11, 12). The addition of point of care ultrasound (POCUS) technology in the NICU could result in more accurate renal NIRS readings as the relative position of the sensor in relation to the kidney can be checked with a simple bedside ultrasound performed by a trained neonatal POCUS provider.

Lastly, it is important to consider implications of artifact and loss of signal. There have been no published articles that have discussed how frequently artifact or loss of signal occurs with neonatal renal tissue oxygenation monitoring. Clinically relevant artifact may occur during dislodgement of a sensor, such as during turning of a baby. These artifacts are typically readily apparent with non-physiologic, large changes between adjacent measurements or prolonged periods of missing data. However, distinguishing the natural variability in renal oxygenation measures from artifact may be challenging. From a research perspective, multiple different techniques to remove artifact have been attempted. In one study, unexplained dips or peaks that were 30% different between two data points were ignored and a manual review of the data was performed to pick the 1 h with the most high quality data for analysis (13). Other research techniques to minimize artifact from cerebral oxygenation data may be adapted for renal oxygenation data. Future standardization for removing suspected artifactual data is needed.

### Normal Values in Term Neonates

**Figure 2:**
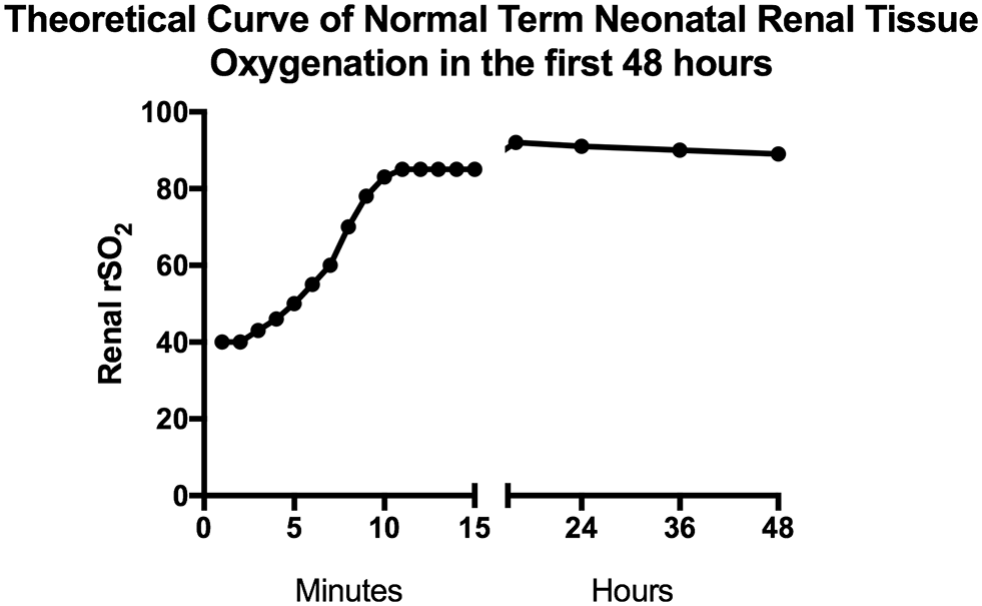
Estimated normal term neonatal renal tissue oxygenation in the first 48 h. This figure depicts predicted normal values of renal tissue oxygenation in term babies during the first 48 h of life based on studies done by Montaldo and Bailey (16, 17). The first 15 min of data are mean values from the Montaldo et al. study and the remaining data are median values from the Bailey et al. study.

After nearly 15 years of published studies on neonatal tissue oxygenation, estimates of normal neonatal renal tissue oxygenation levels are becoming clearer. Normal renal tissue oxygenation values in the neonate are dependent on numerous factors but the three primary driving factors appear to be gestational age, chronologic age or post-menstrual age, and hemoglobin status (2, 14–16). In Supplemental Table 1, we attempt to summarize neonatal publications presenting renal tissue oxygenation data. Starting in the delivery room, Montaldo et al. studied renal NIRS values in the first 15 min after birth in term newborns (17). They found that renal tissue oxygenation starts off in the 40% range immediately after birth and as SpO_2_ improves to normal levels during the first 10 min of life, renal tissue oxygenation also improves to the mid 80% range. In the first 48 h of age after the delivery room, renal NIRS values in term newborns continue to increase to about 90% and slowly decrease as renal blood flow improves and oxygen utilization increases**—**fitting with the expected normal renal developmental physiology (16). As healthy term newborns are most often discharged in the first 48 h of life, it remains to be seen what typical renal tissue oxygenation is in the days, weeks, and months until the age of 2 years when renal function is perceived to have matured. The combination of data from these two studies is depicted in Figure 2 as the theoretical curve for healthy term newborn renal tissue oxygenation in the first 48 h of life.

### Normal Values in Preterm Neonates

However, in preterm infants, the trajectory is likely much more complex and depends significantly on gestational age at birth and degree of illness. For the very low birthweight and extremely low birthweight neonates, the first factor is ensuring placement of the relatively large neonatal NIRS sensor and adherence in a >70% humidified isolette so that a consistent renal measurement can be obtained. After acquisition of a consistent reading, the next challenge is identification of the multiple factors affecting renal tissue oxygenation in a critically ill preterm infant. Some specific factors critical to account for in future studies in the low birth weight preterm population include gestational age at birth, growth for gestational age and birth weight, chronological age and status of the ductus arteriosus.

**Figure 3:**
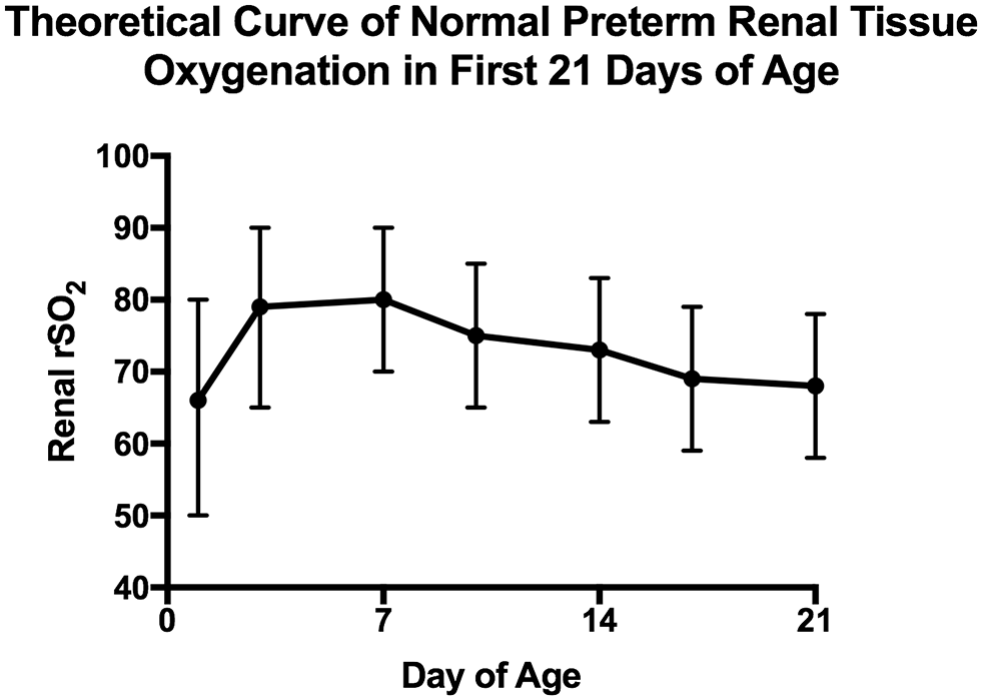
Estimated normal preterm neonatal renal tissue oxygenation in the first 21 days. This figure depicts predicted normal values of renal tissue oxygenation in preterm babies during the first 21 days of life based on studies done by Richter and McNeil (15, 18). For the first 48 h, data combined from all 4 groups of the Richter et al. study are expressed as median and interquartile range while for days 3–21 data are extrapolated from the McNeil et al. study that were presented as mean and standard deviation.

There are a few small studies describing consistent monitoring of preterm renal tissue oxygenation with generation of “normal” values. First, it appears that in the first 48 h, renal tissue oxygenation does not start off as high as is seen in term infants and may be more reflective of poor renal perfusion than high oxygen utilization (18). Richter et al. looked at 80 preterm neonates with gestational age between 25 and 29 weeks and monitored renal tissue oxygenation intermittently during the first 48 h. They found that the median renal tissue oxygenation ranged from 63 to 72% dependent on maternal medication exposures with an interquartile range of 48–87%, suggestive of significant variability. In another study of 14 “clinically stable” preterm newborns between 24 and 36 weeks, McNeil et al. found that over the first 21 days, renal tissue oxygenation starts off in the mid 80% range and trends to the mid 60% range by the third week of age (15). Figure 3 represents a theoretical curve of normal renal tissue oxygenation in preterm newborns during the first 21 days of age based on these two studies. Further information is needed on normal values of renal tissue oxygenation in healthy stable preterm newborns as they progress through their NICU stay to define normal ranges based on both gestational age and post-menstrual age.

### Other Tissue Oxygenation Metrics

In addition to renal tissue oxygen saturation levels, other tissue oxygenation metrics have been reported including fractional tissue oxygen extraction, variability in oxygenation, and cerebral to renal oxygenation ratios. Renal fractional tissue oxygen extraction (rFTOE) is a reflection of oxygen consumption by the kidney and is calculated as (Systemic oxygen saturation**—**Renal tissue oxygen saturation)/(Systemic oxygen saturation). The cerebral to renal oxygenation ratio (CROR) compares renal oxygen levels, which are more sensitive to changes in cardiac output, to brain oxygen levels, which under normal conditions are better autoregulated with relatively stable metabolic demands. Similarly, the degree of variability in renal oxygen saturation is anticipated to be greater than that of cerebral oxygen saturation, with the potential to indicate ongoing changes in an infant's clinical condition. The optimal measures to characterize renal function and detect AKI have yet to be determined. In the following section, we will focus on specific groups of infants and interventions that have been shown to significantly affect renal tissue oxygenation status.

## Renal Tissue Oxygenation in Term Subgroups

### Congenital Heart Disease, Cardiac Surgery, and Extracorporeal Membrane Oxygenation (ECMO)

The population of infants with congenital heart disease (CHD) may be at increased risk for poor systemic perfusion, making monitoring of renal tissue oxygenation with NIRS especially useful to evaluate changes in somatic blood flow. In the pre-operative period, one study demonstrated that renal saturation levels were significantly lower in patients with hypoplastic left heart syndrome (HLHS), tricuspid atresia, or pulmonary atresia with intact ventricular septum (64 ± 11%) compared to controls without CHD (85 ± 6%, *p* = 0.009) during the first 72 h of postnatal life, while compensatory renal fractional tissue oxygen extraction (rFTOE) was significantly higher (31 ± 8 vs. 14 ± 4%, *p* = 0.007) (11). A separate study of 33 infants with transposition of the great arteries or HLHS also confirmed decreased renal saturation levels in the pre-operative period of 60 ± 8% (19). Renal saturation levels do not seem to be significantly affected by the specific type of ductal-dependent congenital cardiac lesion. Over the first 72 h of life, renal saturation decreased for both right and left-sided cardiac lesions which corresponds with decreasing pulmonary vascular resistance and increasing pulmonary blood flow at the expense of systemic blood flow (11). Renal oxygenation monitoring with NIRS would be particularly beneficial during this critical time period of hemodynamic change.

The clinical benefit of monitoring renal saturations in infants with CHD in the pre-operative period has been described. In a historical cohort study, Johnson et al. found that routine pre-operative cerebral and renal NIRS monitoring in patients with HLHS reduced the need for invasive therapies with no difference in mortality or duration of hospital stay (20). Another specific case of HLHS was described (21), where renal NIRS measures decreased from 80 to 50%, providing early indication of decreased systemic blood flow even before other typical clinical findings such as decreased urine output, rising creatinine, elevated lactate, or change in respiratory status. This case highlights the potential value of pre-operative NIRS monitoring of renal oxygenation to guide clinical care in the infant with CHD.

Furthermore, cardiac surgery-associated acute kidney injury (CSA-AKI) is a severe and common complication occurring in 36–52% of infants undergoing cardiac surgery and is associated with increased mortality, prolonged length of hospital stay, ventricular dysfunction, and increased risk of chronic kidney disease (22–24). These infants with CHD are at risk for compromised renal perfusion not only due to their underlying heart condition, but also due to prolonged periods on cardiopulmonary bypass and post-operative low cardiac output syndrome. Limited studies have demonstrated that decreasing renal saturation levels during cardiac surgery and in the post-operative period are associated with AKI, mortality, need for renal replacement therapy, and prolonged ICU stay (25–28). Interventions to improve renal saturation levels may improve outcomes, and further research to substantiate the benefit of renal oxygenation monitoring in the cardiac population is warranted.

A related population of infants who may benefit from monitoring of renal oxygenation are those term infants requiring ECMO. Neonates on ECMO for cardiorespiratory failure typically are at risk for significant shifts in regional blood flow, including blood flow to the kidneys. In addition to alterations in regional blood flow and a patient's underlying disease, the development of AKI during ECMO support may also be related to inflammatory or hormonal factors (29). In a study of babies with congenital diaphragmatic hernia on ECMO, decreasing renal NIRS values were associated with declining urine output and preceded changes in mean arterial blood pressure. A renal saturation of >76% was predictive of adequate urine output (>1 ml/kg/h), with 90% sensitivity and 86% specificity (AUC 0.96) (30). Interventions to improve urine output after a decline in renal saturation levels may avert more severe kidney injury. Additional prospective studies are needed for infants on ECMO support to determine the potential role of renal NIRS monitoring to prevent kidney injury.

### Hypoxic-Ischemic Encephalopathy (HIE)

Neonates with HIE after birth asphyxia are also at significant risk for AKI. Renal perfusion may be impaired from a severe or long-standing antenatal insult combined with ongoing postnatal ischemia and exposure to nephrotoxic medications. For infants with HIE, the development of AKI has been independently associated with prolonged mechanical ventilation, longer hospital course, and brain injury on magnetic resonance imaging (31, 32). Moreover the effect of therapeutic hypothermia for HIE on renal perfusion has not been well-studied. As the kidney is less well-autoregulated than the brain, it is not surprising that renal saturation levels are decreased during cooling (mean 72 ± 9%), which corresponds with the decreased cardiac output, lower heart rate, and peripheral vasoconstriction achieved during cooling (33). During the rewarming period, renal saturations increased back to baseline (87 ± 6%) and renal FTOE decreased as heart rate and cardiac output increased (33, 34). In a single-center study of 38 infants undergoing cooling for HIE, 39% developed AKI and had higher renal saturation levels throughout the cooling period compared to those without AKI (*p* < 0.01). In this study, renal saturation >75% by 24–48 h of life predicted AKI with a sensitivity of 79% and specificity of 82%, potentially reflecting lower oxygen extraction by an injured kidney (33). Detection of an abnormally high renal saturation during cooling would allow clinicians to intervene with kidney protective therapies such as targeting higher blood pressure goals for improved renal perfusion and eliminating nephrotoxic medications. Further research is necessary to evaluate additional therapies to prevent AKI with the targeted use of renal saturation monitoring in this high-risk population of infants with birth asphyxia and HIE.

### Growth Restriction

An interesting population for further evaluation of renal tissue oxygenation monitoring is in growth restricted neonates. Children and adults with a history of fetal growth restriction or low birth weight have been noted in several studies to have a higher risk for CKD (35). However, very few studies have focused on the kidney health of these growth restricted or low birth weight neonates while they are in the NICU. A better understanding of how these infants utilize oxygen in the NICU and early in life may explain the later development of hypertension and CKD. Terstappen et al. sought to evaluate how neonates with intrauterine growth restriction utilize oxygen in the first 3 days after birth compared to a healthy population (13). They conducted an observational study to compare nine control preterm infants to seven preterm infants with fetal growth restriction. They placed renal neonatal sensors shortly after birth and verified placement with a renal ultrasound, ultimately recording continuous data for 72 h after birth. There were differences in maternal and neonatal characteristics between the two groups, with the most significant difference being a consistently higher hemoglobin level in the growth restricted group. With regards to tissue oxygenation, at 3 h when sensors were placed, the two groups had equal tissue oxygenation in the mid to high 80% range. However, soon after birth, the growth restricted group had significantly higher readings in the low 90% range while the control preterm group was in the low to mid 80%. Interestingly, renal FTOE was higher in the control group and lower in the growth restricted group. Renal artery blood flow was similar suggesting that oxygenation differences were due to underlying renal development or physiology. Ultimately it is difficult to conclude without larger numbers of patients if the difference in renal tissue oxygenation is simply due to the hemoglobin difference or truly a difference in oxygen consumption by the kidney. The only other study to evaluate renal tissue oxygenation in growth restricted neonates did not utilize a control group, but found that neonates with an early diagnosis of growth restriction during pregnancy had higher renal FTOE after birth compared to those with a late antepartum diagnosis. The presence of small renal tissue oxygenation abnormalities in these two studies suggest the need for larger and more complete studies that evaluate renal tissue oxygenation, renal blood flow, kidney size, and kidney function in growth restricted neonates. A better understanding of how intrauterine growth restriction results in functional neonatal kidney oxygenation abnormalities may inform NICU treatment and long-term follow-up needs for this population at higher risk for hypertension and development of CKD.

### Surgery and Anesthesia

The use of renal tissue oxygenation monitoring during anesthesia and surgery has been extensively described in the neonatal cardiac surgery population (36). However, only a few studies have looked at non-cardiac surgeries and the use of renal NIRS monitoring. The first group to be evaluated was pediatric patients undergoing laparoscopic abdominal surgery with the theory that potentially high intraabdominal pressures may result in reduced blood flow to the kidneys and renal hypoxia (37). In 29 patients with a mean age of 22 months (number of neonates not detailed), no significant differences in renal tissue oxygenation were found. The investigators concluded that renal hypoxia does not occur if age appropriate intra-abdominal pressures are used. In another study, Beck et al. prospectively evaluated 19 neonates undergoing abdominal surgery (38). In this detailed study, they found that although not statistically significant, renal tissue oxygenation decreased during surgical procedures when abdominal organs were re-introduced into the abdominal cavity (CDH repair, gastroschisis repair, and omphalocele repair). This was consistent with a previous CDH study in which Conforti et al. also found decreases in renal NIRS values at the time of repair (39). The acute decreases in renal tissue oxygenation are likely caused by decreased renal blood flow as intraabdominal pressure acutely increases. Intraoperative renal NIRS monitoring thus may serve as a guide to the need for intravascular volume resuscitation or the need for vasopressor support in neonates who do not have recovery of renal tissue oxygenation after the surgical repair is completed. Finally, Koch et al. studied a combination of over 20 preterm and term neonates undergoing surgery with renal NIRS monitoring (40). In this study, 34 infants received boluses of Ringer's acetate. There were significant increases in renal tissue oxygenation at 5, 15, and 30 min post bolus (~ 5, 25, and 35% increases, respectively). They also noted that in 4 neonates who received epidural boluses of bupivacaine that renal tissue oxygenation decreased significantly at 5, 15, and 30 min post bolus (~ 15, 30, and 25% decreases, respectively). The authors ultimately concluded that renal NIRS monitoring during surgery may play a significant role given the high rate of detected decreases and increases in renal tissue oxygenation with certain interventions that would not otherwise have been detected. We suggest that renal NIRS monitoring be considered in all neonatal surgical cases so that periods of renal hypoxia may be detected and interventions frequently used during surgery can then be evaluated from a renal oxygenation perspective.

## Renal Tissue Oxygenation in Preterm Subgroups

### Patent Ductus Arteriosus (PDA)

Preterm infants are at particular risk for decreased renal perfusion due to the persistence of a hemodynamically significant PDA. Left-to-right shunting through a PDA may result in decreased end organ perfusion. Several NIRS monitoring studies have investigated the effect of the PDA on renal saturation measures. Underwood et al. found a decreased renal saturation <43% in preterm infants in the first 4 days of life predicted treatment with indomethacin or surgical ligation with 77% sensitivity and 83% specificity (41). In the current era of more conservative management of a PDA with less intervention for ductal closure in the first few days of life, NIRS monitoring may have greater benefit at over a week of life when differentiation of a significant PDA is more critical for guiding management. In a contemporary cohort of preterm infants monitored at about a week of life, renal saturation <66% was associated with a significant PDA by echocardiogram (sensitivity 81% and specificity 77%), while association with cerebral saturation was not statistically significant (42). In contrast, other investigators did not find a correlation between size or significance of the PDA and renal tissue oxygenation (43, 44), although discrepancies may be explained by different parameters used for determining echocardiographic PDA significance. Establishing the clinical utility of renal saturation monitoring to better identify infants with a hemodynamically significant ductus will be of critical importance to select which neonates will benefit from ductal closure.

NIRS monitoring may also be useful during treatment of the PDA. Several investigators have documented an increase in renal saturation following indomethacin treatment of a PDA, indicating closure of the ductus (21, 41). However, cyclooxygenase inhibitors such as indomethacin and ibuprofen transiently reduce renal perfusion, and an associated decrease in renal saturation measures has been demonstrated in about one third of treated infants (45). Ibuprofen treatment for ductal closure is less likely to decrease renal oxygenation as measured by NIRS compared to indomethacin treatment (46). This finding is consistent with research documenting the more frequent occurrence of oliguria and increasing creatinine in infants treated with indomethacin compared to ibuprofen for PDA closure (47, 48). Acetaminophen, another drug used to treat a significant PDA, has not yet been investigated with respect to its effects on renal oxygenation, although it does not seem to significantly affect cerebral oxygenation (49). As the cumulative nephrotoxic effects of pharmacologic agents in the preterm infant are being realized, it will be important to assess and optimize renal oxygenation during medical management of a PDA and determine renal oxygenation thresholds or changes that may indicate successful treatment.

### Anemia and Transfusions

Preterm infants are at significant risk for anemia and are likely to undergo transfusions of packed red blood cells during their hospital course. Several investigators have demonstrated an increase in renal tissue oxygenation by 18–22% and a decrease in renal FTOE by 20–30% over the course of a transfusion, indicating enhanced tissue oxygenation after transfusion (14, 50, 51). Pre-transfusion hematocrit did not correlate with renal saturation levels, suggesting that hematocrit alone is a poor predictor of renal tissue oxygenation (50). However, other investigators have found renal FTOE to be inversely correlated with hematocrit level (52). Increased renal FTOE may better identify infants with insufficient tissue oxygen delivery who could benefit from a transfusion before becoming clinically symptomatic. Further research into optimal transfusion thresholds based on regional tissue oxygenation is ongoing.

### Acute Kidney Injury (AKI)

**Figure 4:**
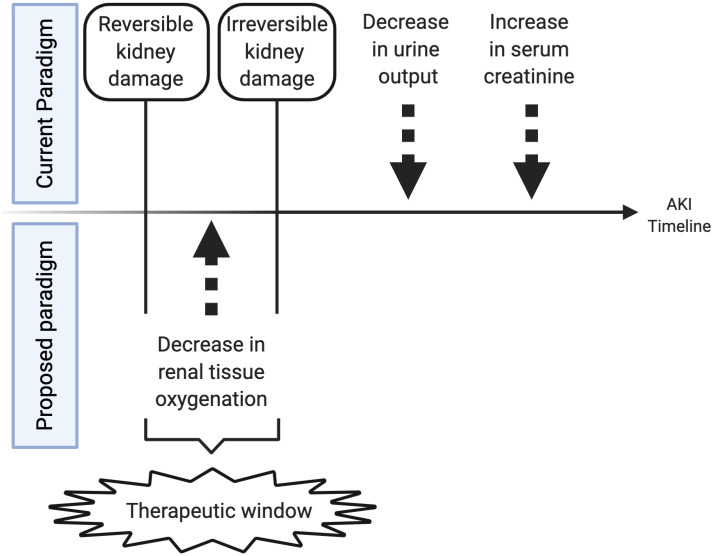
Therapeutic window created by monitoring renal tissue oxygenation. This figure shows the theoretical timeline of changes in a patient with acute kidney injury. Initially tissue oxygenation changes occur followed by a later decrease in urine output and an increase in serum creatinine. Changes in urine output and serum creatinine may signal permanent tissue injury, while changes in tissue oxygenation may reflect an earlier time period when renal ischemia is still reversible and responsive to fluid management, transfusions, inotropic support, or medication administration that may resolve ongoing injury.

While using renal NIRS to determine optimal timing of transfusions is important, the ultimate goal of continuous renal tissue oxygenation monitoring in preterm infants in the NICU would be to prevent AKI. With the current markers of AKI changing 12–48 h after irreversible damage has already occurred, oxygenation changes that occur well before that time may represent a therapeutic window when specific clinical changes or therapies could reverse or prevent injury (Figure 4). Although multiple neonatal studies in cardiac surgery patients have shown that low renal tissue oxygenation correlates with AKI, the type of AKI in preterm patients may be much different than the typical hypoxia reperfusion injury that explains cardiac surgery-associated AKI, and thus renal tissue oxygenation changes may be much different. Preterm AKI likely is highly variable in its origin and includes prerenal, intrarenal, and post-renal causes but the common final pathway may be low tissue oxygenation secondary to hypoperfusion and hypoxia. Bonsante et al. were the first to show that preterm neonatal renal tissue oxygenation changes correlate with future AKI (53). In 128 infants born <32 weeks gestational age and monitored with NIRS, they detected 12 cases of AKI (~10% as defined by serum creatinine > 1.3 mg/dL after the 1st day), and in these patients, low renal tissue oxygenation was significantly associated with developing AKI. After adjusting for possible confounding factors, low renal tissue oxygenation (80 ± 9.5 vs. 69.7 ±11.3%, *p* < 0.001) on the 1st day of life remained associated with high peak serum creatinine on day 2–7. This study was limited with renal tissue oxygenation data only available for the 1st day of age and no information about other nephrotoxic medications the babies may have received. Despite these limitations, this study verifies that a decrease in renal tissue oxygenation prior to increases in serum creatinine are detectable in extremely preterm infants. Significant questions still remain about the exact time frame of tissue oxygenation changes before urine output decreases and serum creatinine increases and if tissue oxygenation correlates to urinary proteomic and metabolomic biomarkers of injury. Further studies are needed to answer these questions.

## Future Directions

A growing body of literature has supported the clinical utility of renal tissue oxygenation monitoring in specific populations of neonates with a high risk of immature or abnormal renal function. However, a significant concern remains about whether or not non-invasive NIRS monitoring of renal tissue oxygenation will have a place in routine assessment of neonatal renal function in both healthy and sick neonates. Although measurement of serum creatinine has significant drawbacks in neonates, it still remains the gold standard assessment of estimated glomerular filtration rate (eGFR). Future techniques of assessing eGFR with serum cystatin C, measuring other urine biomarkers, or counting glomerular number with MRI may play a role in both assessment of current kidney function and prediction of future kidney risk. Further studies on renal tissue oxygenation are needed in all neonatal populations to see if there is a correlation to our current best markers of eGFR, serum creatinine and cystatin C. Furthermore, long-term studies are needed to correlate measures of neonatal renal tissue oxygenation with childhood and adult kidney function. With our current knowledge of renal tissue oxygenation, use of real-time NIRS monitoring is best suited in the populations of neonates and conditions mentioned in this review rather than for estimating renal function. However, significant research still needs to be conducted to better understand the complex relationship between oxygen delivery and oxygen extraction by the kidneys, as well as concurrent cerebral and systemic oxygenation. While population-based norms for renal oxygen saturation have been reported, specific thresholds associated with AKI and anticipated temporal and developmental changes remain as existing knowledge gaps. Moreover, changes in renal perfusion may be reflected by alterations in renal tissue oxygenation, but the extent to which these hemodynamic changes impact kidney function may be quite variable. The physiology of acute and chronic kidney injury may be informed by both renal oxygen saturation measures and renal oxygen extraction data. NIRS monitoring of renal oxygenation will also be critical to evaluate the impact of various therapeutic interventions to preserve kidney function and reduce neonatal AKI. Aside from these research advances, future goals should also include efforts to standardize clinical monitoring protocols, better synchronize data collection of tissue oxygenation with other patient parameters, and develop improved sensor interfaces to more precisely target the kidney and protect neonatal skin. Continued evidence-based research with renal tissue oxygenation monitoring will help achieve the goal of improved neonatal outcomes.

## Author Contributions

VC and MH have both made substantial contributions to writing this review article and have approved the submitted version.

## Conflict of Interest

The authors declare that the research was conducted in the absence of any commercial or financial relationships that could be construed as a potential conflict of interest.
