# Supplementary material for: Renal Tissue Oxygenation Monitoring—An Opportunity to Improve Kidney Outcomes in the Vulnerable Neonatal Population
Source: Front Pediatr. 2020 May 14;8:241. doi: 10.3389/fped.2020.00241 (PMC7247835; doi:10.3389/fped.2020.00241)
Supplement: Supplementary file 1 [file Table_1.docx]

| **Author** | **Study Goal** | **Device** | **Gest Age**  **@ birth** | **N** | **Age** | **Length of Monitoring** | **Pre Intervention RrSO2** | **Post Intervention RrSO2** | **Notes** |
| --- | --- | --- | --- | --- | --- | --- | --- | --- | --- |
| Montaldo^1^ | Tissue oxygenation of term infants during transition | Equanox 7600 | 39 weeks | 61 | 15 min after birth | 15 minutes | 85 |  |  |
| Bailey^2^ | Tissue oxygenation in healthy term newborns | Invos 5100c | 37-42 weeks | 41 | 1-2 days | 48 hours | 88-94 |  | Decrease from 12 hours to 36 hours |
| Richter^3^ | Effect of maternal drugs on tissue oxygenation | Invos 5100c | 28 +/- 2 | 42 | 0-48h | 2 hours | 67 (56-79) |  | No effect of maternal antihypertensive drugs |
| McNeill^4^ | Normal tissue oxygenation in preterm infants | Invos 5100c | 29-34 weeks | 12 | 0-21 days |  | Day 1 = 88  Day 7 = 82  Day 14 = 75  Day 21 = 70 |  | Decrease as older  Variability outside of +/- 15 of baseline 8% of the time in a 24 hours period  Hgb correlation r=0.703 (p<0.005) |
| Elsayed^5^ | Normal tissue oxygenation values in preterm infants |  | 30 +/- 3 wks | 32 | 31 +/- 14d | unclear | Mean = 79  10^th^ % = 73  90^th^ % = 87 |  |  |
| Petrova^6^ | Tissue oxygenation during hypoxic episodes | Invos 5100c | 25-33 weeks | 10 | 1-2 weeks | 2-3 hours | 72 +/- 5 |  | Decreased RrSO2 with mild hypotension |
| Aktas^7^ | Effect of blood transfusions on tissue oxygenation in preterm neonates | Sensmart100 | 30 +/- 2.3 wks | 35 | 17 +/- 19 days | 30-60 min before  24 hours after | 70.7 (median)  49-85 (range) | 72.7 (median)  45-87 (range) |  |
| Dani^8^ | Effect of transfusions on tissue oxygenation | Invos 5100c | Avg 27 +/- 2 | 15 | 32 days +/- 23 | 60 min pre - 60 min post | 64 pre tx | 84 post tx |  |
| Chock^9^ | PDA and tissue oxygenation | Invos 5100c | 27 +/- 2 | 47 | 5-7d | 24 hours prior to echo | hsPDA 61 +/- 3  PDA 70 +/- 3  Unknown 72 +/- 2 |  | hsPDA significantly different |
| van der Laan^10^ | Effect of PDA on tissue oxygenation | Invos 5100c | 28 +/- 2 | 11 | 3-6 days | 3 hours | Closed DA 66 (56-75)  Open DA 55 (44-66)  hsPDA 62 (45-74) |  | No significant differences |
| Guzoglu^11^ | Tissue oxygenation of preterm infants treated with oral ibuprofen | Invos 5100c | 27-29 +/-2 weeks | 30 | 3 (3-7) days | unclear | 57-60 | PDA 60 | No difference in those with hsPDA and no significant differences after ibuprofen |

1. Montaldo P, De Leonibus C, Giordano L, De Vivo M, Giliberti P. Cerebral, renal and mesenteric regional oxygen saturation of term infants during transition. *J Pediatr Surg* 2015, **50**(8)**:** 1273-1277.

2. Bailey SM, Hendricks-Munoz KD, Mally P. Cerebral, renal, and splanchnic tissue oxygen saturation values in healthy term newborns. *Am J Perinatol* 2014, **31**(4)**:** 339-344.

3. Richter AE, Schat TE, Van Braeckel KN, Scherjon SA, Bos AF, Kooi EM. The Effect of Maternal Antihypertensive Drugs on the Cerebral, Renal and Splanchnic Tissue Oxygen Extraction of Preterm Neonates. *Neonatology* 2016, **110**(3)**:** 163-171.

4. McNeill S, Gatenby JC, McElroy S, Engelhardt B. Normal cerebral, renal and abdominal regional oxygen saturations using near-infrared spectroscopy in preterm infants. *J Perinatol* 2011, **31**(1)**:** 51-57.

5. Elsayed YN, Louis D, Ali YH, Amer R, Seshia MM, McNamara PJ. Integrated evaluation of hemodynamics: a novel approach for the assessment and management of preterm infants with compromised systemic circulation. *J Perinatol* 2018, **38**(10)**:** 1337-1343.

6. Petrova A, Mehta R. Regional tissue oxygenation in association with duration of hypoxaemia and haemodynamic variability in preterm neonates. *Arch Dis Child Fetal Neonatal Ed* 2010, **95**(3)**:** F213-219.

7. Aktas S, Ergenekon E, Ozcan E, Aksu M, Unal S, Hirfanoglu IM*, et al.* Effects of blood transfusion on regional tissue oxygenation in preterm newborns are dependent on the degree of anaemia. *J Paediatr Child Health* 2019.

8. Dani C, Pratesi S, Fontanelli G, Barp J, Bertini G. Blood transfusions increase cerebral, splanchnic, and renal oxygenation in anemic preterm infants. *Transfusion (Paris)* 2010, **50**(6)**:** 1220-1226.

9. Chock VY, Rose LA, Mante JV, Punn R. Near-infrared spectroscopy for detection of a significant patent ductus arteriosus. *Pediatr Res* 2016, **80**(5)**:** 675-680.

10. van der Laan ME, Roofthooft MT, Fries MW, Berger RM, Schat TE, van Zoonen AG*, et al.* A Hemodynamically Significant Patent Ductus Arteriosus Does Not Affect Cerebral or Renal Tissue Oxygenation in Preterm Infants. *Neonatology* 2016, **110**(2)**:** 141-147.

11. Guzoglu N, Sari FN, Ozdemir R, Oguz SS, Uras N, Altug N*, et al.* Renal and mesenteric tissue oxygenation in preterm infants treated with oral ibuprofen. *J Matern Fetal Neonatal Med* 2014, **27**(2)**:** 197-203.
